# Inhibitory Effect of *Elaeagnus umbellata* Fractions on Melanogenesis in α-MSH-Stimulated B16-F10 Melanoma Cells

**DOI:** 10.3390/molecules26051308

**Published:** 2021-03-01

**Authors:** Ji-Hyun Lee, Bori Lee, Yong-Deok Jeon, Hyun-Woo Song, Young-Mi Lee, Bong-Joon Song, Dae-Ki Kim

**Affiliations:** 1 Department of Immunology and Institute of Medical Science, Jeonbuk National University Medical School, 20, Geonji-ro, Deokjin-gu, Jeonju-si 54907, Jeollabuk-do, Korea; jihyunsh1211@naver.com; 2 Department of Oriental Pharmacy, College of Pharmacy and Wonkwang-Oriental Medicines Research Institute, Wonkwang University, Iksan 54538, Jeonbuk, Korea; leebori1004@naver.com (B.L.); cristblack@naver.com (H.-W.S.); ymlee@wku.ac.kr (Y.-M.L.); 3 Department of Korean Pharmacy, Woosuk University, 443 Samnye-ro, Samnye-eup, Wanju-Gun 55338, Jeollabuk-do, Korea; ydjeon1211jh@woosuk.ac.kr; 4 Department of Food Science and Biotechnology, Wonkwang University, Iksan 54538, Jeonbuk, Korea; twinf1@hanmail.net

**Keywords:** *Elaeagnus umbellata*, skin-whitening, antioxidant, melanogenesis, α-MSH

## Abstract

When skin is exposed to UV radiation, melanocytes produce melanin. Excessive melanin production leads to skin pigmentation, which causes various cosmetic and health problems. Therefore, the development of safe, natural therapeutics that inhibit the production of melanin is necessary. *Elaeagnus umbellata* (EU) has long been widely used as a folk medicinal plant because of pharmacological properties that include anti-ulcer, antioxidant, and anti-inflammatory properties. In this study, we investigated the antioxidant activity and melanogenesis inhibitory effects of EU fractions in B16-F10 melanoma cells. EU fractions showed a dose-dependent increase in antioxidant activity in radical scavenging activity. In addition, we evaluated the effect of EU fractions on tyrosinase activity and melanogenesis in α-melanocyte-stimulating hormone-induced B16-F10 melanoma cells. EU was noncytotoxic at 12.5–50 μg/mL. EU fractions effectively inhibited tyrosinase activity and melanogenesis, suppressed the phosphorylation of CREB and ERK involved in the melanogenesis pathway, and down-regulated expression of melanogenesis-related proteins. Interestingly, the anti-melanogenesis effect was most effective at a concentration of 50 μg/mL EU, and the effects of the fractions were superior to those of the extract. Therefore, our study suggests that EU has potential as a safe treatment for excessive pigmentation or as a natural ingredient in cosmetics.

## 1. Introduction

Skin color in humans depends on the amounts of melanosomes genetically present, and the degree to which these melanosomes are dispersed in the skin and is also affected by the degree of sunlight exposure and environmental pollution [1]. Melanin is a high molecular weight compound found in animals and plants and is an important pigment that determines the eye, skin, and hair color of humans. Melanogenesis refers to the production process of melanin, a major cause of pigmentation in human skin. Melanin can protect the skin from harmful UV radiation, but excessive melanin production can lead to esthetic problems, including melasma and freckles, as well as health problems, including post-inflammatory hyperpigmentation [2].

Melanin plays a crucial role in protecting the skin from various stimuli, such as UV, reactive oxygen species (ROS), and α-melanocyte stimulating hormone (α-MSH) [3]. When the skin is exposed to UV radiation, ROS is produced, and the skin cells generate excess melanin. This generated ROS destroys single and double stranded DNA and attacks protein and lipid molecules [4]. Melanin and antioxidant enzymes, such as peroxidase, glutathione, and catalase, neutralize the generated ROS to maintain a particular level [5,6]. However, uncontrollable excessive ROS production leads to a variety of problems, such as cancer [7]. Melanocytes are located in the basal layer of the epidermis and are modulated by tyrosinase. These cells produce melanin through melanogenesis, and factors such as microphthalmia-associated transcription factor (MITF) and tyrosinase-related protein (TRP-1) are involved in this process [8]. Tyrosinase is involved in the pathway by which L-tyrosine becomes 3,4-dihydroxyphenylalanine (DOPA), which is then converted to dopaquinone from which melanin is synthesized. Therefore, tyrosinase is an important factor that regulates melanogenesis in skin cells.

Recently, to solve the esthetic problems caused by pigmentation and pigmentary disorder caused by excess melanogenesis, many medical and cosmetic industries have focused on tyrosinase activity inhibitors [9]. Various chemicals such as arbutin, kojic acid, hydroquinone, and ascorbic acid, which are melanin synthesis inhibitors, are used as skin-whitening agents [10]. However, these substances do not penetrate the skin well and over an extended period of use can have side effects such as skin irritation, inflammation, itchiness, and pigmentation [11]. Therefore, there is a need to develop effective whitening agents that can safely treat and prevent the hyperpigmentation of human skin without irritation.

*Elaeagnus umbellata* (EU) is a plant that is widely distributed throughout Asia and has long been used as a folk medicinal plant because of associated pharmacological effects. Traditionally, the flowers and seeds of EU have been used for the treatment of respiratory diseases, including those affecting the heart and lungs, whereas the leaves have been used as a tonic and for treating intestinal diseases [12,13]. In addition, EU is used as a muscle relaxant, analgesic, anti-ulcer, anti-diabetic, and antipyretic agent [14]. EU ingredients have anti-cancer [15], antioxidant, and anti-inflammatory effects [16], and EU extract has an effect on skin whitening and wrinkle improvement [17]. However, studies regarding the effect of skin whitening by branches and leaves in the EU fractions have not yet been conducted. Therefore, the purpose of this study was to observe the in vitro antioxidant activity and melanogenesis inhibition of branch and leaf derived EU fractions on melanin producing B16-F10 murine melanoma cells and to confirm the potential of branch and leaf derived EU as a cosmetic skin-whitening agent.

## 2. Materials and Methods

### 2.1. Materials and Reagents

Primary antibodies for mouse anti-MITF (#sc-25386), tyrosinase (#sc-15341), TRP-1 (#sc-514900), TRP-2 (#sc-74439), p-ERK (#sc-7383), ERK 1/2 (#sc-514302), and β-actin (#sc-47778) were obtained from Santa Cruz Biotechnology (Santa Cruz, CA, USA). The primary anti-bodies for mouse anti-cyclic AMP (cAMP) response element-binding protein (CREB) (8763) (#9198S) and CREB (D76D11) (#4820S) were purchased from Cell Signaling Technology (Beverly, MA, USA); all primary antibodies were used at 1:1000 dilutions. Secondary mouse antibody for primary antibodies was purchased from Enzo Life Sciences (Farmingdale, NY, USA) and used at 1:5000 dilutions. Fetal bovine serum (FBS), Dulbecco’s modified Eagle’s medium (DMEM), and penicillin/streptomycin were purchased from Gibco BRL (Carlsbad, CA, USA). Most chemicals, including α-MSH, 3-(4,5-dimethylthiazol-2-yl)-2,5-diphenyl tetrazolium bromide (MTT), L-DOPA, 1,1-diphenyl-2-picryl-hydrazy (DPPH), 2, 2-azino-bis (3-ethylbenzothiazoline-6-sulfonic acid (ABTS), arbutin, and mushroom tyrosinase were purchased from Sigma-Aldrich (St. Louis, MO, USA).

### 2.2. Preparation of the EU Extract and Fractions as Well as Sample Preparation

Branches and leaves (1:1) of EU were provided by the Jeonbuk Institute for Food Bioindustry (Jeonju, Jeonbuk, Korea). EU (50 g) was ground using a blender, and EU powders were extracted with 70% ethanol for 24 h at room temperature. The solvent was sterile filtered through a standard sieve (Advantec MFS, Taipei, Taiwan) and evaporated under reduced pressure using a rotary evaporator (EYELA, N-1110, Tokyo, Japan). The extract was lyophilized using a freeze dryer (OPERON, FDU-8606, Gimpo, Korea) to remove residual ethanol and obtain 2.4 g (yield, 4.8%) of EU 70% ethanol extract (EUEE) powder. EUEE (2.4 g) was suspended in distilled water and was partitioned with ethyl acetate (EUEA, 0.19 g) or butanol (EUBu, 0.91 g) fractions. All extracts and fractions were stored at 4 °C until use.

### 2.3. High Performance Liquid Chromatography (HPLC) Analysis

**Figure 1:**
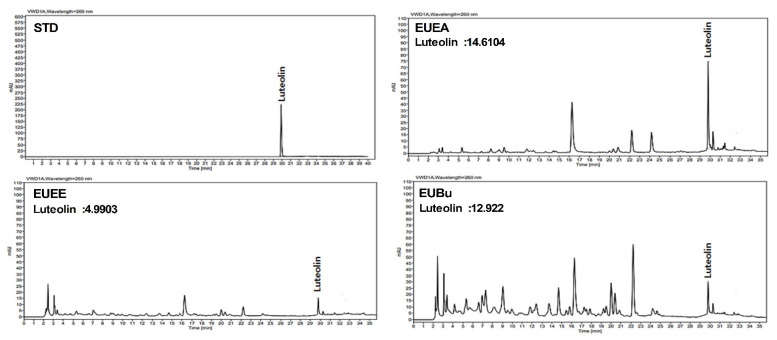
HPLC analysis of EUEE, EUEA, and EUBu. HPLC chromatograms of standard compound (STD) and EU extract and fraction samples. The peak in the HPLC chromatogram indicates luteolin.

Chromatographic analyses were conducted using an Elite Lachrom HPLC-DAD system equipped with a UV detector (Hitachi HighTechnologies Co., Tokyo, Japan). The UV detector at 260 nm and analytical YMC ODS-AM (5 mM, 4.6 × 250 mM) column (Kyoto, Japan) were used for quantification based on the internal standard method. The column temperature was 35 °C, and the mobile phase flow rate was 1 mL/min. The eluent was a gradient of solvent (A) = 0.1% acetic acid in water and solvent (B) = 0.1% acetic acid in acetonitrile. The run time was 35 min, and the gradient was as follows: (A)/(B) = 88/12 (0 min) → (A)/(B) = 78/22 (0–18 min) → (A)/(B) = 72/28 (18–28 min) → (A)/(B) = 62/38 (28–35 min). HPLC analysis is shown in Figure 1.

### 2.4. Cell Culture

Murine melanoma B16-F10 cell line (CRL-6475) was purchased from the American Type Culture Collection (ATCC, VA, USA). Cells were cultured in DMEM supplemented with 10% heat-activated FBS and 1% streptomycin (100 μg/mL)/penicillin (100 units/mL). B16-F10 cells were incubated in humidified 5% CO_2_ at 37 °C.

### 2.5. Cell Viability Assay

The cytotoxicity of the samples was evaluated using the MTT assay. B16-F10 melanoma cells (1 × 10^4^ cells/well) were cultured in a 96-well plate and incubated with various concentrations of extract and fractions (12.5, 25, 50, and 100 μg/mL) or arbutin (100 μg/mL) for 24 h at 37 °C. After incubation, 500 μg/mL MTT solution was added to the wells, and the plate was further incubated for 4 h at 37 °C. Then, the media of each well was removed, and 200 μL dimethyl sulfoxide (DMSO) was added to solubilize the formazan crystals. The absorbance of the plate was detected at 570 nm on a microplate reader (Synergy HTX Multi-Mode Reader, BioTek, Winooski, VT, USA).

### 2.6. DPPH Radical Scavenging Activity

The DPPH radical scavenging activity of the EU extract and fractions was confirmed by measuring the reducing power of DPPH radicals in each sample. Various concentrations (12.5, 25, and 50 μg/mL) of EU extract and fractions or 20 μg/mL of ascorbic acid (positive control) at 500 μL were added to 2 mL of 0.15 mM DPPH solution. The solution was vortexed for 10 s to ensure thorough mixing and was incubated for 30 min at room temperature in the dark. The absorbance of the mixture was then measured at 517 nm using a microplate reader (Biotek, Winooski, VT, USA).

### 2.7. ABTS Radical Scavenging Assay

The ABTS radical scavenging solution was prepared by mixing 7.4 mM of ABTS solution and 2.6 mM of potassium persulfate at a ratio of 1:1 (pH 7.4), and reacting the solution at room temperature for 24 h. The ABTS solution was then adjusted to an absorbance of 0.70 ± 0.03 at 732 nm. Each 10 µL sample was reacted with 190 µL of ABTS solution for 10 min at room temperature, and absorbance was measured at 732 nm using a microplate reader (Biotek, Winooski, VT, USA).

### 2.8. Mushroom Tyrosinase Inhibition Activity

To analyze the inhibitory activity of tyrosinase, which plays a crucial role in melanogenesis, mushroom tyrosinase and L-DOPA substrate were used. First, 150 µL of 0.1 M phosphate buffer (pH 6.8), 20 µL of 5 mM L-DOPA solution, and 20 µL of the various concentrations of EU extract and fractions were sequentially added, and 100 µL of 250 U/mL mushroom tyrosinase was added to initiate the reaction. The mixtures were incubated at 37 °C for 10 min and the absorbance was measured at 475 nm using a microplate reader (Biotek, Winooski, VT, USA). The tyrosinase inhibition activity was calculated using the following formula:

Inhibition activity (%) = (1 − (sample OD-blank OD)/(control OD-blank OD)) × 100

### 2.9. Determination of Cellular Tyrosinase Activity

B16-F10 melanoma cells were seeded at a density of 1 × 10^5^ cells/well in six-well plates containing 2 mL of DMEM. Six groups were established: control group, non-treated; α-MSH group, 100 nM α-MSH only; arbutin group, α-MSH and arbutin (10 mM); EUEE group, α-MSH and EUEE extract (12.5, 25, and 50 μg/mL); EUEA group, α-MSH and EUEA fractions (12.5, 25, and 50 μg/mL); and EUBu group, α-MSH and EUBu fractions (12.5, 25, and 50 μg/mL). Six-well plates were incubated overnight at 37 °C in a 5% CO_2_ incubator. Then, cells were exposed to α-MSH (100 nM) for 48 h and treated with various concentrations of EUEE, EUEA, and EUBu or arbutin (10 mM) for 24 h. Cells were washed with phosphate-buffered saline (PBS) three times and lysed. The lysate was centrifuged at 16,000× *g* for 20 min. Next, the protein content of each lysate was determined using the BCA Protein Assay Kit (Thermo Scientific, Vantaa, Finland). Protein levels were then adjusted to 20 μg in all samples. Lysates (100 μL) containing 2.5 mM of L-DOPA in 0.1 M phosphate buffer was transferred to wells in a 96-well plate and incubated for 1 h at 37 °C. The absorbance of the lysates was measured at 475 nm using a microplate reader (Biotek, Winooski, VT, USA). The intercellular tyrosinase activity was calculated using the following formula:

Tyrosinase activity (%) = (OD_s_ − OD_b_)/Control × 100

OD_s_ represents the absorbance of the sample and OD_b_ represents the absorbance of the control. Arbutin was used as the standard.

### 2.10. Measurement of Melanin Contents

To determine the amount of melanin in B16-F10 cells, these were seeded in a 24-well plate at a density of 2 × 10^4^ cells/well and incubated for 24 h in DMEM with 10% FBS. B16-F10 cells were pretreated with various concentrations of EUEE, EUEA, and EUBu (12.5, 25, and 50 μg/mL) and positive control arbutin (10 mM) for 1 h, then reacted with 100 nM of α-MSH for an additional 72 h. Afterward, cells were harvested after washing with PBS. The pellet obtained by centrifugation (12,000× *g*, 10 min) was lysed in 1N NaOH containing 10% DMSO at 90 °C for 30 min. The melanin content of each well was measured at 405 nm using a microplate reader (Biotek, Winooski, VT, USA). To determine the amount of melanin, the total amounts produced during the experiment were regarded as the standard (100%), and the inhibition rate of each extract and fraction treatment group was determined in proportion to this standard.

### 2.11. Western Blot Analysis

The B16-F10 murine melanoma cells (1 × 10^6^ cells/well) were cultured in 6 cm dishes and incubated overnight at 37 °C, and cells were pretreated with α-MSH (10 µM) for 24 h. Then, cells were treated with various concentrations (12.5, 25, and 50 μg/mL) of EU extract and fractions or 100 μg/mL arbutin for 24 h. Cells were harvested with PBS then centrifuged at 12,000× *g* for 15 min at 4 °C. After removing the supernatant, cells were lysed using ice-cold Pro-prep protein lysis buffer with the addition of 1% protease and phosphatase inhibitors, and kept on ice for 40 min. The fractionated proteins were quantified using the Bradford protein assay. Equal amounts of proteins (25 μg) were loaded and separated by 8% or 10% sodium dodecyl sulfatepolyacrylamide gel electrophoresis and transferred to a polyvinylidene fluoride (PVDF) membrane. The PVDF membranes were blocked with 5% skim milk in Tris-buffered saline (TBS) with 0.1% Tween 20 (TBS-T) buffer for 1 h at room temperature, and incubated with specific primary antibodies against p-CREB, CREB, p-ERK, ERK, MITF, TRP-1, TRP-2, tyrosinase, and β-actin overnight at 4 °C. Afterward, the membranes were rinsed with TBS-T buffer three times for 10 min and reacted for 2 h with horseradish peroxidase-conjugated secondary antibody at room temperature. Membranes were rerinsed with TBS-T buffer five times and proteins were detected with enhanced chemiluminescence solution. The band images were obtained using a Davinch-In vivo and western imaging system (Davinch-K, Seoul, Korea).

### 2.12. Statistical Analysis

All values are presented as means ± S.E.M. (standard error of the mean) of at least three independent experiments and GraphPad Prism software 5.0 (GraphPad, San Diego, CA, USA) was used for statistical analysis. One way analysis of variance followed by Bonferroni post hoc analysis was used to compare the statistical differences between groups. A *p*-value less than 0.05 (*p* < 0.05) was considered statistically significant.

## 3. Results

### 3.1. HPLC Analysis of EU

The final yields (%) of EUEE, EUEA, and EUBu were 4.8%, 0.18%, and 1.82%, respectively based on the fresh weight. HPLC was used to analyze the content of luteolin, an indicator compound of EU extract and fractions. Compared with the peak of the standard component, the peak of luteolin in the EU extract and fractions was confirmed. The concentration of luteolin in EUEE, EUEA, and EUBu was 499.03, 1948.05, and 994.00 mg/mL, respectively (Figure 1).

### 3.2. The Effect of EU on Antioxidant Activity and Tyrosinase Inhibitory Activity

**Figure 2:**
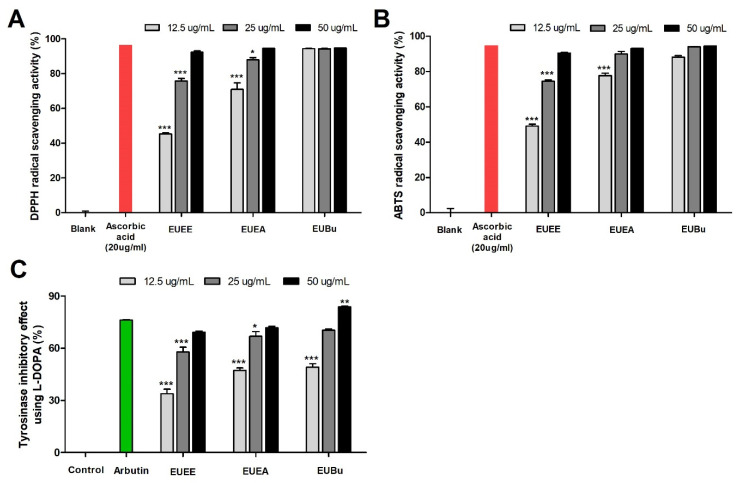
Antioxidant activities and mushroom tyrosinase inhibition activity of EU. Radical scavenging activity of DPPH (**A**) and ABTS (**B**) of EU extract and fractions. Ascorbic acid (20 μg/mL) was used as a positive control. The tyrosinase inhibitory effect using L-DOPA of EU extract and fractions (**C**). Arbutin (10 mM) was used as a positive control. All graphs show per centages of control, and data are expressed as means ± S.E.M. (n = 3). Statistical significance was set at * *p <* 0.05, ** *p <* 0.01 and *** *p* < 0.001 when compared with the ascorbic acid or arbutin group.

The antioxidant activities of the various concentrations of EUEE, EUEA, and EUBu were measured using the DPPH/ABTS assay. The DPPH and ABTS radical scavenging activities of EUEE, EUEA, and EUBu increased in a dose-dependent manner (Figure 2A,B), with ascorbic acid as a positive control. The DPPH and ABTS radical scavenging activities at 50 μg/mL of the EU extract and fractions were both higher, showing similar results to the positive control, ascorbic acid. To measure the inhibitory effect of melanogenesis-mediated enzymes in the EU extract and fractions, a mushroom tyrosinase inhibition assay was performed (Figure 2C) with L-DOPA as a substrate under cell-free conditions. The results showed that tyrosinase activity was inhibited by the EU extract and fractions in a dose-dependent manner. In particular, the tyrosinase inhibitory activity of 50 μg/mL of EU extract and fractions showed similar results to arbutin. Therefore, the tyrosinase inhibitory effect of EU is related to the oxidation of L-DOPA to DOPA quinone in melanogenesis. Overall, the EU fractions had a greater antioxidant and tyrosinase inhibitory effect than that of the extract. In addition, the EU fractions, especially EUBu, began to exhibit similar effects to the positive control group at a dose of 25 μg/mL.

### 3.3. The Effect of EU on B16-F10 Cell Viability

**Figure 3:**
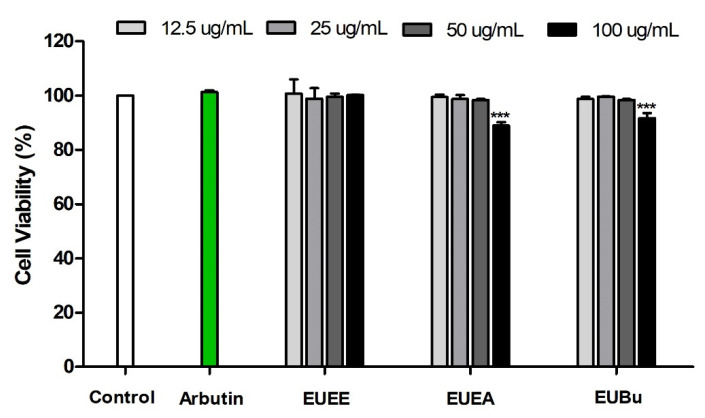
Cell viability of EU. Cell viability in B16-F10 melanoma cells of EUEE, EUEA, and EUBu. All graphs show percentages of control, and data expressed as means ± S.E.M. (n = 3). Statistical significance was set at *** *p <* 0.001 when compared with the control.

To assess the cell cytotoxicity of EU, B16-F10 melanoma cells were treated with various concentrations (12.5, 25, 50, and 100 µg/mL) of EU extract and fractions for 24 h, and cell viability was analyzed by MTT assay. The results are shown as the percentage of cell viability relative to the control. The EU extract and fractions were not cytotoxic in the tested concentration range of 12.5–50 µg/mL to B16-F10 cells. However, cytotoxicity was confirmed in B16-F10 cells at a concentration of 100 μg/mL of the EU fractions (EUEA and EUBu) (Figure 3). Therefore, subsequent experiments were conducted using concentrations of 12.5, 25, and 50 µg/mL of EU extract and fractions.

### 3.4. The Effect of EU on Melanin Synthesis

**Figure 4:**
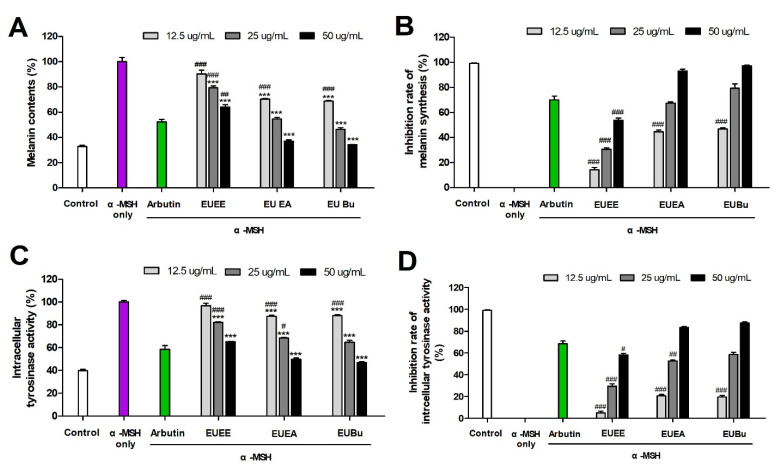
Melanin synthesis and cellular tyrosinase activities of EU. Melanin contents of EU extract and fractions in B16-F10 melanoma cells (**A**). Inhibition rate of melanin synthesis of EU extract and fractions in B16-F10 melanoma cells (**B**). Intracellular tyrosinase activity of EU extract and fractions in B16-F10 melanoma cells (**C**). Inhibition rate of intracellular tyrosinase activity of EU extract and fractions in B16-F10 melanoma cells (**D**). Results (**A**,**C**) are presented as percentages based on the α-MSH group. Results (**B**,**D**) are presented as percentages based on the control group. Data are expressed as the means ± S.E.M. (n = 3). Statistical significance was set at *** *p <* 0.001 when compared with the α-MSH-stimulated B16-F10 cells, and ^#^ *p <* 0.05, ^##^ *p <* 0.01, and ^###^ *p <* 0.001 compared with the arbutin group.

To determine the anti-melanogenic effect of EU, the inhibitory effect of EU extract and fractions on melanin synthesis were examined in B16-F10 cells. The B16-F10 cells were treated with the EU extract and fractions at 12.5, 25, and 50 μg/mL or with arbutin at 10 mM for 1 h and were then stimulated with α-MSH (100 mM) for 72 h. The melanin content increased by 305.44% when stimulated with α-MSH compared with that of the control (Figure 4A). However, the EU-treated groups at a concentration of 50 µg/mL had significantly reduced melanin content compared with that of the α-MSH-only stimulated group, and the results were similar to those of the control group. In addition, compared with that of the arbutin positive control, the EU fractions at a concentration of 50 µg/mL exhibited a lower level of melanin production. Consistent with these results, as the concentrations of EU extract and fractions increased, the inhibition rate of melanin synthesis in B16-F10 cells increased (Figure 4B). In particular, the inhibition rate of melanin synthesis in the EU fractions at a concentration of 50 μg/mL was significantly higher than that of arbutin. Therefore, the EU fractions had a significant dose-dependent inhibitory effect on melanin synthesis in B16-F10 melanoma cells.

### 3.5. The Effect of EU on Melanin Synthesis and Cellular Tyrosinase Activity

We confirmed the effect of EU extract and fractions on intracellular tyrosinase activity involved in the mechanism of melanogenesis in B16-F10 melanoma cells. All groups, except the control, were stimulated with α-MSH (100 nM) for 48 h then treated with various concentrations of EU extract and fractions (12.5, 25, and 50 μg/mL) or arbutin (10 mM) for 24 h. As a result, EU extract and fractions significantly inhibited α-MSH-induced tyrosinase activity in B16-F10 cells in a dose-dependent manner (Figure 4C). Intracellular tyrosinase activity increased by 251.00% when stimulated with α-MSH compared with that of the control. However, the EU-treated groups at a concentration of 50 µg/mL significantly decreased tyrosinase activity compared with that of the α-MSH-only stimulated group. Thus, tyrosinase activity was inhibited in a dose-dependent manner by EU treatment in B16-F10 cells, and the effect was particularly strong at a concentration of 50 μg/mL. The inhibition of tyrosinase activity by EUEE at a concentration of 50 μg/mL was lower than that of arbutin (85.13%), whereas inhibition of tyrosinase activity of EU fractions (EUEA and EUBu) was higher than that of arbutin (121.99 and 128.11%, respectively). Consistent with these results, as the concentrations of EU extract and fractions increased, the inhibition rate of intracellular tyrosinase activity in B16-F10 cells increased (Figure 4D).

### 3.6. The Effect of EU on Melanogenesis-Related CREB and ERK Proteins Expression

**Figure 5:**
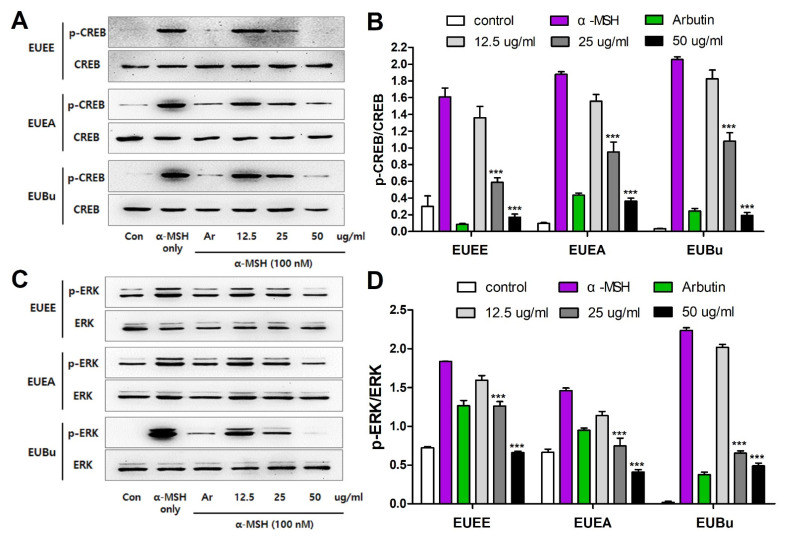
Inhibitory effect of EU on melanogenesis-related CREB and ERK protein expression. Representative western blotting band image of p-CREB and CREB (**A**) and p-ERK and ERK (**C**). Using the GelQuantNET program, the relative intensity of the western blotting band is shown in the following bar graphs: p-CREB/CREB (**B**) and p-ERK/ERK (**D**). Data are expressed as the mean ± S.E.M. (n = 3). Statistical significance was set at *** *p <* 0.001 when compared with the α-MSH-stimulated B16-F10 cells.

Melanogenesis protects human skin from various external stimuli, and various mechanisms are involved in this process. α-MSH, a hormone involved in the first stage of melanogenesis, stimulates melanocytes to phosphorylate CREB and ERK, inducing the synthesis of down-signal proteins associated with melanogenesis [18]. In this study, we investigated the melanogenesis-regulated proteins, including CREB and ERK, to determine the melanogenesis inhibitory mechanism of EU on α-MSH-induced melanin synthesis in B16-F10 melanoma cells. As a result, we confirmed that CREB and ERK phosphorylation significantly increased when B16-F10 cells were stimulated with α-MSH. However, when cells were treated with EU extract and fractions, α-MSH-induced phosphorylation was suppressed in a dose-dependent manner. In particular, it can be seen that the EU extract and fractions of 50 μg/mL concentration are the most effective among various treatment concentrations (Figure 5A–D).

### 3.7. The Effect of EU on Melanogenesis-Related MITF, TRP-1, TRP-2, and Tyrosinase Proteins Expression

**Figure 6:**
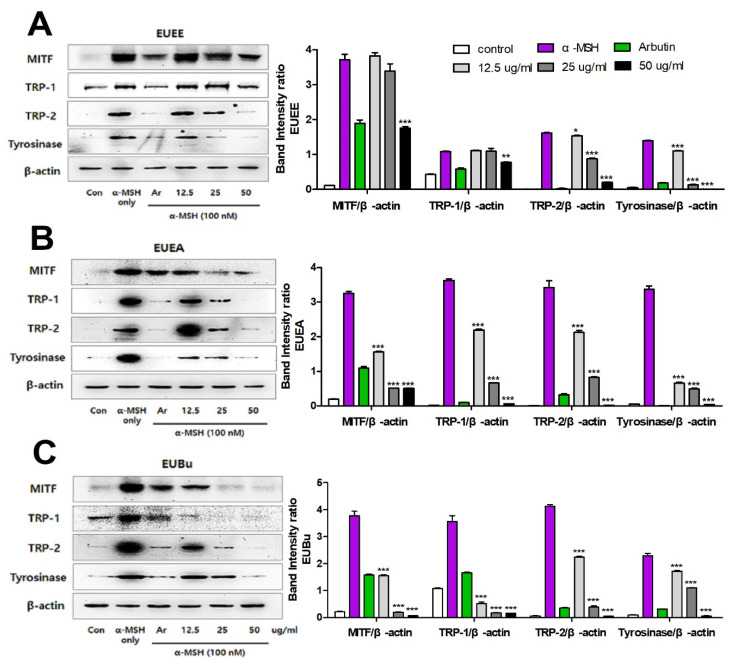
Inhibitory effect of EU on melanogenesis-related MITF, TRP-1, TRP-2, and tyrosinase proteins expression. Representative western blotting band image of MITF, TRP-1, TRP-2, and tyrosinase of EUEE (**A**), EUEA (**B**), and EUBu (**C**). Using the GelQuantNET program, the relative intensity of the western blotting band is shown as the following bar graph; MITF/β-actin, TRP-1/β-actin, TRP-2/β-actin, and tyrosinase/β-actin. β-actin was used as the internal control of western blotting. Data are expressed as the means ± S.E.M. (n = 3). Statistical significance was set at * *p <* 0.05, **p < 0.01, and *** *p <* 0.001 when compared with the α-MSH-stimulated B16-F10 cells.

To investigate whether the EU extract and fractions affect the expression of melanogenesis-related proteins induced by α-MSH, western blotting was performed to assess the expression of MITF, TRP-1, TRP-2, and tyrosinase proteins. Levels of all the melanogenesis-related proteins were significantly increased by α-MSH stimulation, but EU extract and fractions effectively suppressed the expression of MITF, TRP-1, TRP-2, and tyrosinase proteins in α-MSH-stimulated B16-F10 cells (Figure 6). Consistent with the previous results, especially with most of the EU extracts and fractions at 50 μg/mL, expression of these proteins was more inhibited than that found following treatment with positive control arbutin. In addition, the treatment with EU fractions induced a greater effect at the same concentration compared with that of the EU extract.

## 4. Discussion

Melanocytes are located in the base layer of skin, and the degree of melanin synthesis in melanocytes determines the color of human skin [19]. Melanin is a skin pigment protects skin by reducing the amount of ROS generated by harmful ultraviolet rays. When the skin is exposed to UV radiation, α-MSH, which stimulates melanocytes, is activated, and melanocytes produce melanin [20]. However, excessive melanin production can cause pigmentation on the skin, causing esthetic problems.

Binding of α-MSH to melanocyte receptor melanocyte-specific melanocortin-1 receptor (MC1R) activates signaling protein adenylate cyclase and increases the intracellular levels of cAMP [21]. As the level of cAMP in melanocytes increases, the CREB and ERK signaling pathways that promote the production of proteins involved in melanogenesis are activated. [18,22]. This activated CREB and ERK signaling pathway up-regulates expression of MITF and subsequently promotes the expression of TRP-1, TRP-2, and tyrosinase [23]. MITF is a transcription factor for tyrosinase regulation and plays a central role in regulating melanin synthesis, including regulating the proliferation, differentiation, and survival of melanocytes [24]. This protein not only regulates melanogenesis by regulating sub-proteins, including TRP-1, TRP-2, and tyrosinase, but is also involved in regulating cell cycle progression [25]. Taken together, α-MSH-MC1R binding caused by external stimulation, such as UV radiation, mainly activates CREB and ERK signaling pathways in melanocytes, thereby increasing the expression of MITF, TRP-1, TRP-2, and tyrosinase proteins to induce the production of melanin. Therefore, inhibition of this process may result in the inhibition of melanin synthesis in melanocytes.

At present, the various skin whitening agents including hydroquinone and hydrocortisone in use are mostly harmful chemicals, and long-term use can cause side effects, including skin irritation, and itchiness [1,26,27,28]. Therefore, it is necessary to focus on the development of safe and effective natural skin-whitening agents with minimal side effects like EU [29].

In this study, we investigated how EU fractions inhibit melanin synthesis processes and reduce the production of melanin, resulting in the skin whitening effect in B16-F10 melanoma cells.

Since oxidative stress caused by UV radiation induces the deposition of melanin pigments, radical scavenging activity and inhibition of ROS activity are effective in sup-pressing melanogenesis, and natural products with such antioxidant activity can have excellent whitening effects [30,31]. Therefore, we first investigated the antioxidant activity of EU extract and fractions in B16/F10 melanoma cells using DPPH and ABTS assays, which are common and simple methods for analyzing the antioxidant activity of plants in vitro. DPPH is a stable free radical and is used to analyze the radical scavenging activity of hydrophobic compounds using reducing agents, such as ascorbic acid and tocopherol. The ABTS assay can measure radical scavenging activity for hydrophobic and hydrophilic compounds, chain breaking antioxidants, and hydrogen-donating antioxidants [24]. We confirmed the high DPPH and ABTS radical scavenging activities of EU extracts and fractions. In particular, the effects were strongest at a concentration of 50 μg/mL, and the EU fractions had superior antioxidant activity compared with that of the extract.

Tyrosinase is a major enzyme involved in melanogenesis, and many skin-whitening agents are primarily tyrosinase inhibitors. Therefore, we examined the effect of EU fractions on tyrosinase activity, which promotes melanogenesis. We confirmed the inhibitory effect of L-DOPA on cellular tyrosinase activity to determine whether EU ex-tract and fractions directly inhibit tyrosinase activity. As a result, the EU extract and fractions experienced an increase in the tyrosinase inhibitory effect in a dose-dependent manner. In addition, consistent with the above results, EU was most effective at a concentration of 50 μg/mL, and the EU fractions had a greater effect than that of the extract. In particular, the concentration of EUBu (50 μg/mL) had more effect than that of arbutin, a positive control.

Next, we investigated the skin-whitening effect of EU extract and fractions on melanogenesis in α-MSH-induced B16-F10 melanoma cells. We first used the MTT assay to establish a concentration range at which the EU extract and fractions were non-cytotoxic to B16-F10 cells. Our results confirmed that there was no cytotoxicity in the concentration range of 12.5 to 50 μg/mL.

After stimulating B16-F10 cells with α-MSH to induce melanin synthesis, the skin-whitening effect of EU extract and fractions on melanin content and intracellular tyrosinase activity was identified. The patterns of EU extract and fractions on melanin con-tent and intracellular tyrosinase activity showed similar results. Treatment with all EU extracts and fractions showed that melanin content and intracellular tyrosinase activity were dose-dependently suppressed. In particular, at a concentration of 50 μg/mL of EU fractions, more than 50% loss of melanin content and intracellular tyrosinase activity was observed compared with that of the α-MSH-only stimulated group (Figure 4A,C).

CREB and ERK signaling pathways are major signaling pathways that are activated by the stimulation of α-MSH in melanogenesis. Therefore, CREB and ERK, which are involved in various mechanisms, play a key role in the melanin synthesis process. Stimulation of α-MSH in B16-F10 melanoma cells increased the expression of MITF and the melanogenesis-related tyrosinase gene family (TRP-1, TRP-2, and tyrosinase) through the activation of CREB and ERK. In this study, we provide evidence that EU extract and fractions inhibit melanin synthesis as well as the involved upstream signaling pathways, CREB and ERK.

When the levels of TRP-1 and TRP-2 were increased by up-regulated expression of MITF, the expression of tyrosinase was promoted and the melanogenesis-related enzymes produced melanin [18,32]. The expression of melanogenesis-mediated proteins was observed using western blotting to determine the anti-melanogenic effect of EU extract and fractions in B16-F10 melanoma cells. Our results confirmed that the expression of MITF, TRP-1, TRP-2, and tyrosinase proteins significantly increased in the α-MSH-stimulated group. However, treatment with EU extract and fractions down-regulated expression of these melanogenesis-related proteins in a dose-dependent manner. In particular, a significant decrease in expression was observed at a concentration of 50 μg/mL of EU fractions. Our results show that 50 μg/mL of EU fractions inhibited expression of MITF, an important transcription factor, significantly inhibiting tyrosinase expression, and thereby inhibiting melanin production.

Based on these results, we found that 50 μg/mL of EU fractions inhibited the production of α-MSH-stimulated melanin production in B16-F10 melanoma cells by down-regulating the signaling related to melanogenesis based on the excellent antioxidant effect. Therefore, our results suggest that the EU fraction may be a potential candidate for a whitening treatment that effectively controls melanogenesis.

## 5. Conclusions

In this study, we evaluated the antioxidant and anti-melanogenic effects on α-MSH-induced melanogenesis in B16-F10 melanoma cells to confirm the function of EU fractions as skin-whitening agents. In conclusion, we found that EU fractions have strong antioxidant effects and inhibited melanogenesis-related proteins, such as TRP-1, TRP-2, and tyrosinase, by regulating MITF and are therefore valuable natural inhibitors of melanogenesis. Notably, a concentration (50 µg/mL) of EU extract and fractions was required to inhibit melanin synthesis in α-MSH-stimulated B16F10 cells. In addition, we confirmed that EU fractions have superior antioxidant and inhibitory effects on melanin production. Consequently, the EU could be useful for natural-based therapeutic agents for treating skin diseases and as skin-whitening agents in the cosmetics industry.

## Sample Availability

Samples of the compounds are not available from the authors.

## Abbreviations

α-MSH	α-melanocyte stimulating hormone
CREB	cAMP response element binding protein
DOPA	3,4-dihydroxyphenylalanine
EU	Elaeagnus umbellata
EUEE	EU 70% ethanol extract
EUEA	EU ethyl acetate fraction
EUBu	EU butanol fraction
MC1R	melanocyte-specific melanocortin-1 receptor
MITF	Microphthalmia-associated transcription factor
ROS	Reactive oxygen species
TRP	Tyrosinase-related protein
